# Postoperative Refractory Diarrhea After Margin Accentuation of the Superior Mesenteric Artery with Irreversible Electroporation in Pancreaticoduodenectomy

**DOI:** 10.3390/jcm14103568

**Published:** 2025-05-20

**Authors:** Eline-Alice Brys, Filip Gryspeerdt, Nikdokht Rashidian, An Verena Lerut, Pieter Dries, Luís Abreu de Carvalho, Frederik Berrevoet

**Affiliations:** 1 Faculty of Medicine and Health Sciences, Ghent University, 9000 Ghent, Belgium; elinealice.brys@ugent.be; 2 Department of General and HPB Surgery and Liver Transplantation, Ghent University Hospital, 9000 Ghent, Belgium

**Keywords:** pancreatic adenocarcinoma, irreversible electroporation, margin accentuation

## Abstract

**Background**: Pancreatic ductal adenocarcinoma (PDAC) presents a challenge due to its poor prognosis. Irreversible electroporation (IRE) shows promise in improving margin clearance and increasing R0 and R1 indirect resection rates. Although IRE is believed to preserve surrounding tissues, this study aimed to assess postoperative refractory diarrhea as a severe complication and challenge the assumption of consistent tissue preservation. **Methods**: Patients undergoing pancreaticoduodenectomy (PD) with IRE for superior mesenteric artery (SMA) margin accentuation between May 2022 and April 2024 were included. Primary endpoints were diarrhea-related morbidity and mortality; secondary endpoints included R-status, recurrence, and metastases. IRE electrodes were initially positioned circumferentially around the SMA, but this approach was modified to hemi-circumferential placement and applied in six additional patients. **Results**: All five patients (median age 70, 80% female) in the initial cohort developed secretory diarrhea lasting a median of 6 months (IQR 5–6.5), with a median frequency of 5 stools/day (IQR 5–6.5). Two patients (40%) died due to diarrhea-associated cachexia. In contrast, among the six patients treated with the modified technique, patients’ diarrhea resolved within a median of 8 days (IQR 6–10) without need for opioid or advanced antidiarrheal therapy. **Conclusions**: Circumferential IRE for SMA margin accentuation may damage the superior mesenteric plexus and induce severe, prolonged diarrhea. Hemi-circumferential application may mitigate this risk. Larger studies are required to validate these findings and optimize the use of IRE in PD.

## 1. Introduction

Pancreatic ductal adenocarcinoma (PDAC) is the seventh leading cause of cancer death worldwide because of its poor prognosis and high mortality rate. Especially in Western countries, PDAC of the pancreatic head is known as a major upcoming burden of disease [1].

Complete tumor resection with free margins is still the only chance for cure [2,3,4]. This microscopic evaluation is described as the residual tumor (R) classification which includes R0 resection indicating no residual tumor within 1 mm from all margins, R1 indirect resection which indicates the presence of microscopic residual tumor within 1 mm from a margin, R1 direct resection as the presence of tumor at one of the margins, and R2 resection reflecting macroscopic residual tumor [4,5]. It is known that approximately 70% of surgical resection specimens are categorized as R1 direct, which precludes patients’ definitive cure [5].

Recent advancements have developed irreversible electroporation (IRE) as a new perioperative approach with high potential for treating prostate cancer [6] and varied results in palliative therapy of locally advanced pancreatic cancer [7,8,9,10,11]. IRE is now being studied for its effectiveness in enhancing margin clearance in (borderline) resectable pancreatic cancer surgery, with the possibility of increased R0 and R1 indirect resection rates [8,12,13,14]. IRE is a non-thermal ablative treatment that induces apoptosis of tumor cells, particularly those at risk of invading vital structures such as the portal system or the superior mesenteric artery (SMA) by generating an electric field in between paired electrodes. Cellular apoptosis is achieved while preserving the structural integrity of the surrounding tissues [7,11,12,13].

In this article, we aimed to challenge the assumption that tissue preservation is consistently achieved. Animal studies have demonstrated that IRE can induce transient injury to surrounding tissue. In a porcine model, duodenal mucosal damage was observed following IRE in the pancreatic head region [15], while a separate swine study reported pancreatic tissue injury, perivascular fibrosis, and anatomical narrowing of nearby vessels despite maintained perfusion [16]. While these findings are limited to animal models and causality in humans remains unproven, they suggest that surrounding anatomical structures—particularly those in close proximity to the ablation zone—may not be entirely unaffected. In this article, we further challenge the notion that IRE uniformly spares surrounding non-target tissues.

To obtain the highest chance for R0 or R1 indirect resection during pancreaticoduodenectomy (PD), it is often mandatory to dissect the nerve plexus around major arteries such as the SMA and the celiac trunk, as also proposed in recent surgical innovative techniques such as the Triangle operation and the peri-arterial divestment technique [17,18,19]. Recent studies acknowledge postoperative refractory diarrhea as a severe complication of this superior mesenteric plexus dissection [20,21,22,23,24,25]. Because postoperative diarrhea after pancreaticoduodenectomy is associated with a reduced adherence and completion of adjuvant chemotherapy and a lower quality of life, it is known as an important issue in clinical practice [24].

## 2. Materials and Methods

### 2.1. Patient Selection

This retrospective study focused on five pancreatic cancer patients who were surgically treated with pancreaticoduodenectomy using IRE for margin accentuation of the SMA at our tertiary hospital between May 2022 and February 2023. These cases were specifically analyzed due to the development of postoperative refractory diarrhea. These patients represent the first five cases at our institution in which IRE was employed for SMA margin accentuation during pancreaticoduodenectomy.

The primary endpoint of this analysis was the assessment of morbidity and mortality associated with the occurrence of refractory diarrhea postoperatively. Secondary endpoints included R-status at the SMA margin, local recurrence rates, and rates of distant metastasis.

Relevant clinical data about demographic features, medical history, surgical procedure, postoperative morbidity and mortality were collected from the pseudonymized prospectively maintained database. Patients were followed-up after the procedure over a period of 24 months between May 2022 and May 2024.

The following inclusion criteria apply for the use of IRE for margin accentuation during PD. Patients must have a histological diagnosis of PDAC with radiological features of borderline resectable pancreatic head cancer (BRPC) or locally advanced pancreatic head cancer (LAPC) that has been successfully down-staged after administration of neoadjuvant chemotherapy. BRPC is defined as partial encasement of the SMA (<180 degrees) and/or infiltration of the mesenteric–portal axis with possibility of reconstruction. LAPC is described as encasement of the SMA (>180 degrees) and/or encasement of the mesenteric–portal axis without possibility of reconstruction [7]. As described by Kwon and colleagues [8], patients without metastatic disease and presenting features of BRPC or successfully down-staged LAPC with partial encasement of the SMA were considered potential candidates for pancreaticoduodenectomy with IRE SMA margin accentuation. All patients underwent neo-adjuvant chemotherapy treatment with FOLFIRINOX. Following this systemic therapy, patients were restaged with radiological imaging.

### 2.2. Operative Procedure: Margin Accentuation with IRE

**Figure 1:**
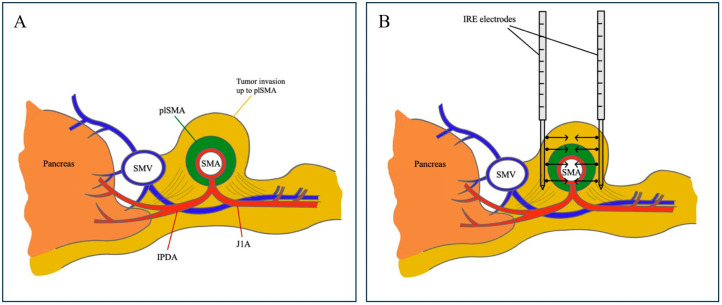
Intraoperative IRE application for SMA margin accentuation. Cross-sectional images at the level of the pancreatic head providing insight into (**A**) the pancreas, superior mesenteric vein (SMV) and superior mesenteric artery (SMA) with inferior pancreaticoduodenal artery (IPDA) and first jejunal branch (J1A). plSMA indicates the superior mesenteric plexus and is situated in the surrounding area of SMA. Tumor cells of the pancreatic head adenocarcinoma invade the mesopancreas up to plSMA. (**B**) displays the circumferential placement of the IRE-electrodes around both the left and right side of the SMA for margin accentuation. Black arrows indicate the direction of the electric current generated by the electrodes.

The delivery of IRE for SMA margin accentuation was performed with NanoKnife (AngioDynamics^®^, Latham, NY, USA). Needle electrodes were positioned circumferentially on both sides of the SMA to directly treat potential tumor cells at this location. At the time of dissection of the pancreatic head, both the left and right side of the SMA were targeted with the electrodes, guided perioperatively by high-definition ultrasound imaging (Figure 1). Each electrode had an effective electric field range of 1–1.5 cm. Electric pulses had a voltage of 1500 V/m and a wavelength of 100 s [11]. Considering the limited length of needle probe exposure, multiple pullbacks were often required. Pulses were administered synchronously with the electrocardiogram. Following a test pulse, treatment was delivered.

**Figure 2:**
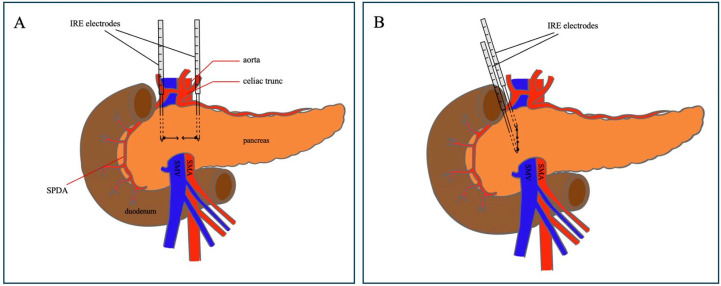
Circumferential and hemi-circumferential placement of the electrodes for IRE application for SMA margin accentuation. (**A**) illustrates the circumferential placement of the IRE-electrodes around both the left and right side of the SMA for margin accentuation. Black arrows indicate the direction of the electric current generated by the electrodes. (**B**) illustrates the protocol adjustment to hemi-circumferential placement of the electrodes on the right side of the SMA. Black arrows indicate the direction of the electric current generated by the electrodes. Abbreviations: SPDA = superior pancreaticoduodenal artery.

In response to the severe complication of postoperative diarrhea in these five initial patients, we revised our surgical protocol to position the electrodes hemi-circumferentially on the right side of the SMA for all subsequent patients undergoing PD with IRE for SMA margin accentuation (Figure 2). This modified hemi-circumferential approach was implemented in six subsequent patients undergoing PD with SMA margin accentuation between August 2023 and April 2024, with follow-up extending through May 2025.

Histopathological assessment was performed on two occasions: initially intraoperatively using frozen section analysis for rapid evaluation of the resected margins, followed by standard histopathological evaluation using hematoxylin and eosin staining. The evaluation of the residual tumor status (R) was then conducted, being based on guidelines outlined by Schlitter and colleagues. R0 resection was defined as a microscopic tumor clearance of >1 mm, R1 indirect resection as a clearance of ≤1 mm, and R1 direct resection as presence of tumor cells at the resected margin [26].

### 2.3. Ethical Approval

This study was approved by the Medical Ethics Committee of Ghent University Hospital under protocol code BC-10695 on 6 October 2021. Ethical approval for the prospectively maintained database was obtained under protocol code BC-08135 on 6 August 2020. Informed consent was obtained from all patients.

### 2.4. Statistics

A descriptive statistical analysis was conducted on all available variables. Continuous variables were represented using median and interquartile range (IQR). Categorical variables were represented using frequencies and percentages. Computations were carried out using the statistical software SPSS (IBM SPSS Statistics, Version 28.0).

## 3. Results

This analysis evaluates five pancreatic cancer patients, four women and one man, median age 70 (IQR 64–71) years, due to the occurrence of refractory diarrhea after margin accentuation of the SMA with IRE. At the time of initial diagnosis, 80% (*n* = 4) were classified as LAPC and 20% (*n* = 1) as BRPC. All five patients (100%) featured tumors situated in the uncinate process with partial encasement of the SMA as observed at their CT imaging, with a median tumor size of 2.5 cm (IQR 2.2–4.3) (Table 1).

Both BRPC (80%, *n* = 4) and LAPC (20%, *n* = 1) patients underwent 12 cycles of FOLFIRINOX-based chemotherapy, as outlined by our institution’s protocol: 40% (*n* = 2) received all 12 cycles in neoadjuvant treatment, while 60% (*n* = 3) received the chemotherapy cycles dispersed across neoadjuvant and adjuvant setting. Following their neoadjuvant treatment, the LAPC cases (80%) showed promising response and were successfully down-staged, rendering them eligible for surgical exploration. Subsequently, all patients (*n* = 5) underwent pancreaticoduodenectomy with margin accentuation of the SMA-margin using IRE.

R0 resection was successfully achieved in 20% (*n* = 1) and R1 indirect resection in 60% (*n* = 3), and 20% (*n* = 1) underwent a R1 direct resection. In the immediate postoperative period, 40% (*n* = 2) required reoperation due to postoperative bleeding associated with immediate anticoagulation therapy administered following venous reconstruction. No mortality was observed within 30 days post-IRE procedure.

In the immediate postoperative period following PD with IRE margin accentuation, all patients (*n* = 5) were closely monitored and provided with enteral nutrition (Table 2). Early postoperative manifestations of malabsorption, including diarrhea and mild dehydration, were observed in all patients (100%). The dosage of pancreatic enzymes was therefore increased; however, diarrhea persisted, occurring a median of 5 times per day (IQR 5–6.5). Despite this adjustment, the continued presence of diarrhea necessitated the addition of loperamide, a mild opioid derivative, to the treatment regimen.

Median length of hospital stay was 14 days (IQR 11.5–18.5). Upon discharge, all patients (*n* = 5) continued to experience diarrhea. At the one-month follow-up consultation, diarrhea remained persistent. The stools were secretory in nature, without blood, mucus, or fat, and diarrhea predominantly occurred postprandially, without nocturnal episodes. All patients (100%) required further adjustments in their treatment regimen, including additional increases in pancreatic enzyme dosages and loperamide.

Two to three months postoperatively, all patients (*n* = 5) sought medical assistance due to ongoing excessive diarrhea, with a median weight loss of 9 kg (IQR 7.5–12). Clinical examination showed dehydration symptoms, such as tachycardia, hypotension, and vertigo. Diagnostic tests, including blood analysis, venous gas analysis, fecal examination, and hydrogen breath tests, were conducted. Except for hypokalemia in three patients (60%), all results were within normal limits, and no pathogens were identified in coprocultures. Notably, one patient (20%) exhibited signs of steatorrhea.

As a result, a secondary treatment plan was implemented to address the persistent diarrhea. This included the administration of synthetic somatostatin octreotide to three patients (60%), and opioid tramadol to one (20%), and anti-hyperlipidemic agent colestyramine along with spasmolytic otilonium for the patient with steatorrhea (20%). After a median treatment duration of 6 months (IQR 5–6.5) with potent antidiarrheal agents, the diarrhea remained present.

At the 24-month follow-up, no local recurrence of disease was observed. Forty percent (*n* = 2) developed distant metastases in the lungs and peritoneum. The overall survival rate was 20% (*n* = 1). Causes of death included progressive clinical decline due to refractory diarrhea and cachexia in two patients (40%), and distant metastatic disease in two patients (40%). The interval between the IRE-procedure and death ranged from 10.2 to 19.6 months, with a median survival time of 15.2 months.

Following the modification of the IRE protocol from circumferential to hemi-circumferential needle placement around the SMA, six patients (two men and four women, median age 68 (IQR 65–72) years) underwent SMA margin accentuation using the adapted technique (Table 3). An R0 resection was achieved in 66.6% (*n* = 4), and 33.3% (*n* = 2) had an R1 indirect resection. All patients (*n* = 6) developed postoperative diarrhea with a median duration of 8 days (IQR 6–10). No patients required opioid analgesia, and diarrhea resolved upon initiation of pancreatic enzyme supplementation. Local recurrence was observed in one patient (16.7%) after a median interval of 7.6 months. Distant metastases developed in two patients (33.3%) with a median time to onset of 9.5 months (IQR 7.1–11.8). At 12 months of follow-up, five patients (83.3%) were alive, and one patient (16.7%) had died due to local recurrence. The time from IRE to death in this patient was 8 months.

## 4. Discussion

Postoperative diarrhea following PD can arise from various factors such as adjuvant chemotherapy or exocrine pancreatic insufficiency [20]. However, in this series, we hypothesize that SMA margin accentuation using IRE may have played a key role in the onset of diarrhea. Given the 1–1.5 cm effective radius of IRE electrodes [11], and the proximity of the superior mesenteric plexus to the SMA, electric pulses may inadvertently damage neural tissue, potentially contributing to refractory diarrhea. A porcine study reported transient duodenal mucosal injury following IRE in the pancreatic head region [15], suggesting that surrounding gastrointestinal structures may be susceptible to stress-related changes. Additionally, another swine model showed post-IRE pancreatic tissue injury with perivascular fibrosis and anatomical narrowing of nearby vessels, despite preserved flow [16]. These findings could reinforce that IRE, while non-thermal, may still result in structural damage to nearby tissues, including vasculature and possibly neural plexuses, which could contribute to postoperative gastrointestinal complications.

Severe diarrhea after PD is often associated with the complete circumferential surgical dissection of the superior mesenteric plexus surrounding the SMA, which is often necessitated due to invasion of the nerve plexus by PDAC [20,21,22,23,24]. Recent surgical techniques, such as the Triangle operation, also insist on complete clearance of the periarterial nervous plexus [17,18,19,20,21]. According to Klotz et al., diarrhea occurs significantly more with the Triangle operation, which involves radical surgical resection of the lymphatic tissue located between the celiac trunk, SMA, and the mesenteric–portal axis [25]. Due to the correlation of diarrhea with a reduced compliance to adjuvant chemotherapy and a diminished quality of life [24], severe postoperative diarrhea is a relevant issue to consider in clinical practice.

After surgical dissection of the SMA, patients typically experience watery diarrhea within two hours postprandially, occurring more than five times daily, and unmanageable with non-opioid antidiarrheal medication [20,21]. Our analysis demonstrated similar findings after SMA margin accentuation with IRE, suggesting that postoperative diarrhea after margin accentuation may stem from disruption of the superior mesenteric plexus.

A possible approach to avoid the induction of diarrhea could be placing the IRE-electrodes hemi-circumferentially on the right of the SMA. Placing the electrodes only on the right side of the SMA might still have its local effect within the field range of 1–1.5 cm, without complete destruction of the nervous plexus. Although some studies suggest that hemi-circumferential surgical dissection does not significantly reduce diarrhea incidence [21,24], our experience shows that after modifying the study protocol, postoperative refractory diarrhea was absent in all subsequent patients.

Currently, there are no significant recommendations for managing postoperative refractory diarrhea following nervous plexus disruption. Inoue and colleagues [24] suggested using opioids in the immediate postoperative period, which we also found somewhat effective in controlling diarrhea after five to seven months, though the diarrhea remained present.

Several limitations should be acknowledged in this study. While initial modification to the IRE technique appears promising, these observations are based on a small sample size, necessitating larger studies to confirm these results and evaluate the broader implications. The observational nature of the study prevents establishing a definitive causal relationship between IRE and postoperative refractory diarrhea. The lack of a standardized quality of life assessment tool and a validated severity scoring system for diarrhea is also acknowledged.

## 5. Conclusions

Circumferential IRE for SMA margin accentuation in pancreaticoduodenectomy may cause severe refractory diarrhea, likely due to potential damage to neural cells within the superior mesenteric plexus. Modifying the technique to hemi-circumferential electrode placement was associated with markedly reduced symptoms and improved postoperative tolerance. While promising, these findings are based on a limited cohort and should be validated in larger prospective studies to guide safe and effective use of IRE in pancreatic cancer surgery.

## Figures and Tables

**Table 1:** Clinical, operative, and postoperative characteristics of patients treated with the initial circumferential IRE approach.

Characteristics	Patients (*n* = 5) *n* (%) Median (IQR)
**Preoperative**	
Sex	
Female	4 (80%)
Male	1 (20%)
Age, years	70 (64–71)
BMI, kg/m^2^	22.7 (21–25.1)
Tumor size, cm	2.5 (2.2–4.3)
Resectability	
LAPC	4 (80%)
BRPC	1 (20%)
**Intra-operative**	
Margin accentuation of SMA with IRE	
Administration frequency	2 (1–2.5)
Additional SMA dissection?	
Yes	1 (20%)
No	4 (80%)
Residual tumor status R	
R0 resection	1 (20%)
R1 indirect resection	3 (60%)
R1 direct resection	1 (20%)
R2 resection	0
ypTNM classification	
ypT4NxM0	2 (40%)
ypT2NxM0	2 (40%)
ypT2N0M0	1 (20%)
**Postoperative**	
Length of in hospital stay, days	14 (11.5–18.5)
30-day mortality	0
Reoperation	2 (40%)
Reason: bleeding after anticoagulation	2
Local recurrence	0
Development of metastases	2 (40%)
Time between IRE and metastases, months	8 (6.9–9.2)
Status at 24-month follow-up	
Dead	4 (80%)
Alive	1 (20%)
Cause of death	
Progressive clinical decline	2 (40%)
Distant metastatic disease	2 (40%)
Time between IRE and death, months	15.2 (10.2–19.6)

BRPC = borderline resectable pancreatic head cancer; LAPC = locally advanced pancreatic head cancer.

**Table 2:** Postoperative refractory diarrhea characteristics.

Characteristics	Patients (*n* = 5) *n* (%) Median (Range)
Stool	
Secretory	5 (100%)
Steatorrhea	1 (20%)
Coproculture	
Positive	0
Negative	5 (100%)
Frequency per day	5 (5–6.5)
Body weight loss ^a^, kg	9 (7.5–12)
Duration after surgery, months	6 (5–6.5)

^a^ = Difference between weight at the time of surgery and weight after secondary antidiarrheal treatment.

**Table 3:** Follow-up outcomes of subsequent patients treated with the modified hemi-circumferential IRE approach for SMA margin accentuation.

Characteristics	Patients (*n* = 6) *n* (%) Median (Range)
R-status	
R0	4 (66.6%)
R1 indirect	2 (33.3%)
R1 direct	0
R2	0
Duration of postoperative diarrhea, days	8 (6–10)
Local recurrence	1 (16.7%)
Time between IRE and recurrence, months	7.6 (7.6–7.6)
Development of metastases	2 (33.3%)
Time between IRE and metastases, months	9.5 (7.1–11.8)
Status at 12-month follow-up	
Dead	1 (16.7%)
Alive	5 (83.3%)
Cause of death	
Local recurrence	1 (16.7%)
Time between IRE and death, months	8 (8–8)

## Data Availability

The original contributions presented in this study are included in the article. Further inquiries can be directed to the corresponding author.

## Abbreviations

The following abbreviations are used in this manuscript:

PDAC	Pancreatic ductal adenocarcinoma
IRE	Irreversible electroporation
PD	Pancreaticoduodenectomy
SMA	Superior mesenteric artery
R	Residual tumor
BRPC	Borderline resectable pancreatic cancer
LAPC	Locally advanced pancreatic cancer
IQR	Interquartile range
